# Prognostic factors for advanced lung cancer patients with do-not-intubate order in intensive care unit: a retrospective study

**DOI:** 10.1186/s12890-022-02042-7

**Published:** 2022-06-24

**Authors:** Chia-I Shen, Shan-Yao Yang, Hwa-Yen Chiu, Wei-Chih Chen, Wen-Kuang Yu, Kuang-Yao Yang

**Affiliations:** 1 Department of Chest Medicine, Taipei Veterans General Hospital, 201, Section 2, Shih-Pai Road, Taipei, 112 Taiwan; 2 School of Medicine, College of Medicine, National Yang Ming Chiao Tung University, 155, Section 2, Linong Street, Taipei, 112 Taiwan; 3 Institute of Clinical Medicine, College of Medicine, National Yang Ming Chiao Tung University, 155, Section 2, Linong Street, Taipei, 112 Taiwan; 4 Department of Internal Medicine, Taipei Veterans General Hospital Hsinchu Branch, Hsinchu County, Taiwan; 5 Institute of Biophotonics, National Yang Ming Chiao Tung University, 155, Section 2, Linong Street, Taipei, 112 Taiwan; 6 Institute of Emergency and Critical Care Medicine, College of Medicine, National Yang Ming Chiao Tung University, 155, Section 2, Linong Street, Taipei, 112 Taiwan; 7 Cancer Progression Research Center, National Yang Ming Chiao Tung University, 155, Section 2, Linong Street, Taipei, 112 Taiwan

**Keywords:** Non-small cell lung cancer, Do-not-intubate, Critical care

## Abstract

**Background:**

The survival of patients with lung cancer undergoing critical care has improved. An increasing number of patients with lung cancer have signed a predefined do-not-intubate (DNI) order before admission to the intensive care unit (ICU). These patients may still be transferred to the ICU and even receive non-invasive ventilation (NIV) support. However, there is still a lack of prognostic predictions in this cohort. Whether patients will benefit from ICU care remains unclear.

**Methods:**

We retrospectively collected data from patients with advanced lung cancer who had signed a DNI order before ICU admission in a tertiary medical center between 2014 and 2016. The clinical characteristics and survival outcomes were discussed.

**Results:**

A total of 140 patients (median age, 73 years; 62.1% were male) were included, had been diagnosed with stage III or IV non-small cell lung cancer (NSCLC) (AJCC 7th edition), and signed a DNI. Most patients received NIV during ICU stay. The median APACHE II score was 14 (standard error [SE], ± 0.66) and the mean PaO2/FiO2 ratio (P/F ratio) was 174.2 (SD, ± 104 mmHg). The APACHE II score was significantly lower in 28-day survivors (survivor: 12 (± 0.98) vs. non-survivor: 15 (± 0.83); *p* = 0.019). The P/F ratio of the survivors was higher than that of non-survivors (survivors: 209.6 ± 111.4 vs. non-survivors: 157.9 ± 96.7; *p* = 0.006). Patients with a P/F ratio ≥ 150 had better 28-day survival (*p* = 0.005). By combining P/F ratio ≥ 150 and APACHE II score < 16, those with high P/F ratios and low APACHE II scores during ICU admission had a notable 28-day survival compared with the rest (*p* < 0.001). These prognostic factors could also be applied to 90-day survival (*p* = 0.003). The prediction model was significant for those with driver mutations in 90-day survival (*p* = 0.021).

**Conclusions:**

P/F ratio ≥ 150 and APACHE II score < 16 were significant prognostic factors for critically ill patients with lung cancer and DNI. This prediction could be applied to 90-day survival in patients with driver mutations. These findings are informative for clinical practice and decision-making.

**Supplementary Information:**

The online version contains supplementary material available at 10.1186/s12890-022-02042-7.

## Background

Cancer patients account for over 15% of all admissions to intensive care units (ICUs) [1, 2]. This proportion continues to rise, as lifespan has been prolonged [3]. In the early 1990s, critically ill patients with cancer were not candidates for ICU admission because of their high mortality rate [4, 5]. However, the concept and practice have changed over the past decades owing to the progress in anticancer treatment and advanced life-support systems [6, 7]. Current evidence shows improved survival in critical care and supports transfer to the ICU of patients with either oncological or hematological malignancies [8]. Lung cancer is the leading cause of death worldwide, accounting for the majority of critically ill cancer patients [2, 9]. The survival of lung cancer patients in the ICU has improved in recent years [10–13]. However, the clinical presentation is diverse among these lung cancer patients, making it difficult to predict their survival during the ICU course [14–16]. Moreover, an increasing number of patients have signed a predefined do-not-intubate (DNI) order before admission to the ICU [17–19]. Our team has shown that non-invasive ventilation (NIV) as a first-line therapy for respiratory failure in lung cancer patients have higher mortality rate compared with those used NIV for post-extubation [20]. However, few studies have focused on the prognostic factors for lung cancer patients with DNI who require intensive care [21]. Whether these patients may benefit from NIV and critical care remains unclear [1, 21]. Here, we designed a retrospective study to evaluate the prognostic factors in patients with lung cancer admitted to the ICU. We hope to find evidence for further decision-making.

## Material and methods

### Patient cohort

This retrospective study was conducted at a tertiary medical center in Taiwan. We focused on the patients admitted to the respiratory ICU. Patients with advanced stage non-small cell lung cancer (NSCLC), including stages III and IV, who had signed DNI orders before admission to the ICU were included. The exclusion criteria were (1) recent surgery related to lung cancer within 30 days, (2) newly diagnosed NSCLC during the ICU course, and (3) intubation or withdrawal of the DNI order during the admission course. The staging system used was the AJCC 7^th^ edition. The patient enrollment duration was between January 1, 2014 and December 1, 2016. The protocol was approved, and informed consent was waived by the institutional review board of Taipei Veterans General Hospital (2020-04-008CC).

### Study variables

We collected patient clinical data, including age, sex, performance status (Eastern Cooperative Oncology Group, ECOG), BMI, and underlying diseases. Comorbidities such as heart failure, cirrhosis, coronary artery disease, chronic obstructive pulmonary disease, previous cerebrovascular accident, and thromboembolic events (including deep vein thrombosis or pulmonary embolism) were defined by a medical chart review. For the patients’ cancer status, we documented their histology type, TNM staging, driver mutations, intracranial condition, previous anticancer treatment lines, and recent treatment. Anticancer treatment lines represented total systemic treatment lines, including the current treatment. Anticancer treatment that occurred during the 30 days prior to ICU admission was defined as recent anticancer treatment. Treatment can be divided into (1) chemotherapy, (2) immunotherapy (with or without chemotherapy), (3) targeted therapy, and (4) radiotherapy. For ICU course evaluation, we documented the date of admission to the ICU and date of discharge from the ICU (including transfer to the ordinary ward, discharge against advice, and death). For the patients’ critical status, we recorded their Glasgow Coma Scale (GCS) score, PaO_2_/FiO_2_ ratio (P/F ratio), and APACHE II score during admission. The reasons for ICU admission were divided into four categories: (1) cancer-related, which included obstructive pneumonia (documented as an infection in the lung secondary to tumor obstruction), respiratory failure due to diffuse lung involvement with cancer, cardiac tamponade, tumor bleeding, neurologic events, and metabolic events; (2) treatment-related complications, including radiation pneumonitis, drug-induced lung toxicity, neutropenia, and other treatment-related complications; (3) infections, such as pneumonia and other infections that are not clearly related to cancer treatment; and (4) underlying comorbidities, such as acute exacerbation of chronic obstructive pulmonary disease and pulmonary edema. We focused on the outcomes of 28-day and 90-day mortality rates.

### Statistical analysis

Pearson’s chi-squared or Fisher’s exact tests were used to compare categorical variables. Continuous variables were compared using the Student’s t-test, for normally distributed variables, and the Mann–Whitney U test for non-normally distributed variables. Kaplan–Meier survival curves were plotted for overall survival. Univariate and multivariate associations between clinical features and outcomes were analyzed using the Cox proportional hazards regression model. Statistical significance was set at *p* < 0.05, and all *p*-values were two-sided. SPSS software (version 21.0) was used for all the analyses.

## Results

### Baseline characteristics

In total, 140 patients (median age, 73 years; age range, 44–99 years) were included in our study (Additional file 1: Figure S1). All patients were diagnosed with stage III or IV non-small cell lung cancer and signed a DNI consent before ICU admission between 2014 and 2016. Approximately 62.1% of all patients were male, and approximately half were ever-smokers. About 42.9% of the patients had driver mutations. The median APACHE II score was 14 (SE, ± 0.66). Nearly 97.9% of all patients received NIV during ICU stay. The mean P/F ratio was 174.2(SD, ± 104 mmHg). A comparison of the basic characteristics of 28-day survivors and non-survivors showed no statistical difference between age, sex, smoking status, and performance status. The APACHE II score was significantly lower in 28-day survivors (survivors: 12 (± 0.98) vs. non-survivors: 15 (± 0.83); *p* = 0.019). The P/F ratio of the survivors was higher than that of non-survivors (survivors: 209.6 ± 111.4 vs. non-survivors 157.9 ± 96.7; *p* = 0.006). Other critical statuses, including arterial blood gas data, lactate levels, and neutropenia, did not differ between survivors and non-survivors. Reasons for ICU admission were similar. Anticancer treatments during the 30 days prior to ICU admission were quite similar in both groups. The baseline characteristics are shown in Table 1.

**Table 1:** ICU basic characteristics (n = 140)

	All patients (n = 140)	28 days Survivors (n = 44)	28 days Non-survivors (n = 96)	*p* Value
Age (Median, range)	73 (44–99)	74 (47–99)	72 (44–93)	0.054
BMI (Median, range)	21 (13–33)	21 (13–32)	21 (15–33)	0.971
Sex				
Male	87 (62.1)	29 (65.9)	58 (60.4)	0.578
Female	53 (37.9)	15 (34.1)	38 (39.6)	
Smoking history				
Ever smoker	76 (54.3)	24 (54.5)	52 (54.2)	1.00
Never smoker	64 (45.7)	20 (45.5)	44 (45.8)	
ECOG				
0–1	62 (44.3)	15 (34.1)	47 (49.0)	0.142
> = 2	78 (55.7)	29 (65.9)	49 (51.0)	
Driver mutation	60 (42.9)	20 (45.5)	40 (41.7)	0.715
Treatment lines	2 (0–10)	1 (0–8)	2 (0–10)	0.142
APACHEII (Median, SE)	14 (± 0.66)	12 (± 0.98)	15 (± 0.83)	0.019
NIPPV^a^	137 (97.9)	43 (97.7)	94 (97.9)	1.000
Recent anticancer treatment				
Nil^b^	52 (37.1)	18 (40.9)	34 (35.4)	0.110
Chemotherapy	38 (27.1)	8 (18.2)	30 (31.3)	
Target therapy	37 (26.4)	16 (36.4)	21 (21.9)	
Radiotherapy	13 (9.3)	2 (4.5)	11 (11.5)	
GCS	14 (3–15)	15 (3–15)	14 (3–15)	0.172
PaO_2_/FiO_2_ (Mean, SD)	174.2 ± 104	209.6 ± 111.4	157.9 ± 96.7	0.006
PaCO_2_	42.5 ± 16.4	41.3 ± 13.8	43.1 ± 17.5	0.542
pH	7.40 ± 0.11	7.41 ± 0.11	7.40 ± 0.01	0.430
Lactate	28.8 ± 24.5	26.2 ± 29	30 ± 22.5	0.499
Neutropenia	7 (5)	1 (2.3)	6 (6.3)	0.433
Reasons of ICU admission				
Sepsis/infection	73 (52.1)	21 (47.7)	52 (54.2)	0.553
Cancer related	40 (28.6)	13 (29.5)	27 (28.1)	
Treatment related	15 (10.7)	7 (15.9)	8 (8.3)	
Underlying disease	12 (8.6)	3 (6.8)	9 (9.4)	

^a^Three patients did not use NIPPV: one infection, one liver failure due to cancer progression, and one IICP due to tumor

^b^”Nil” represents patients who did not receive any anticancer treatment 30 days prior to ICU admission

### 28-day mortality and predictive factors

**Fig. 1:**
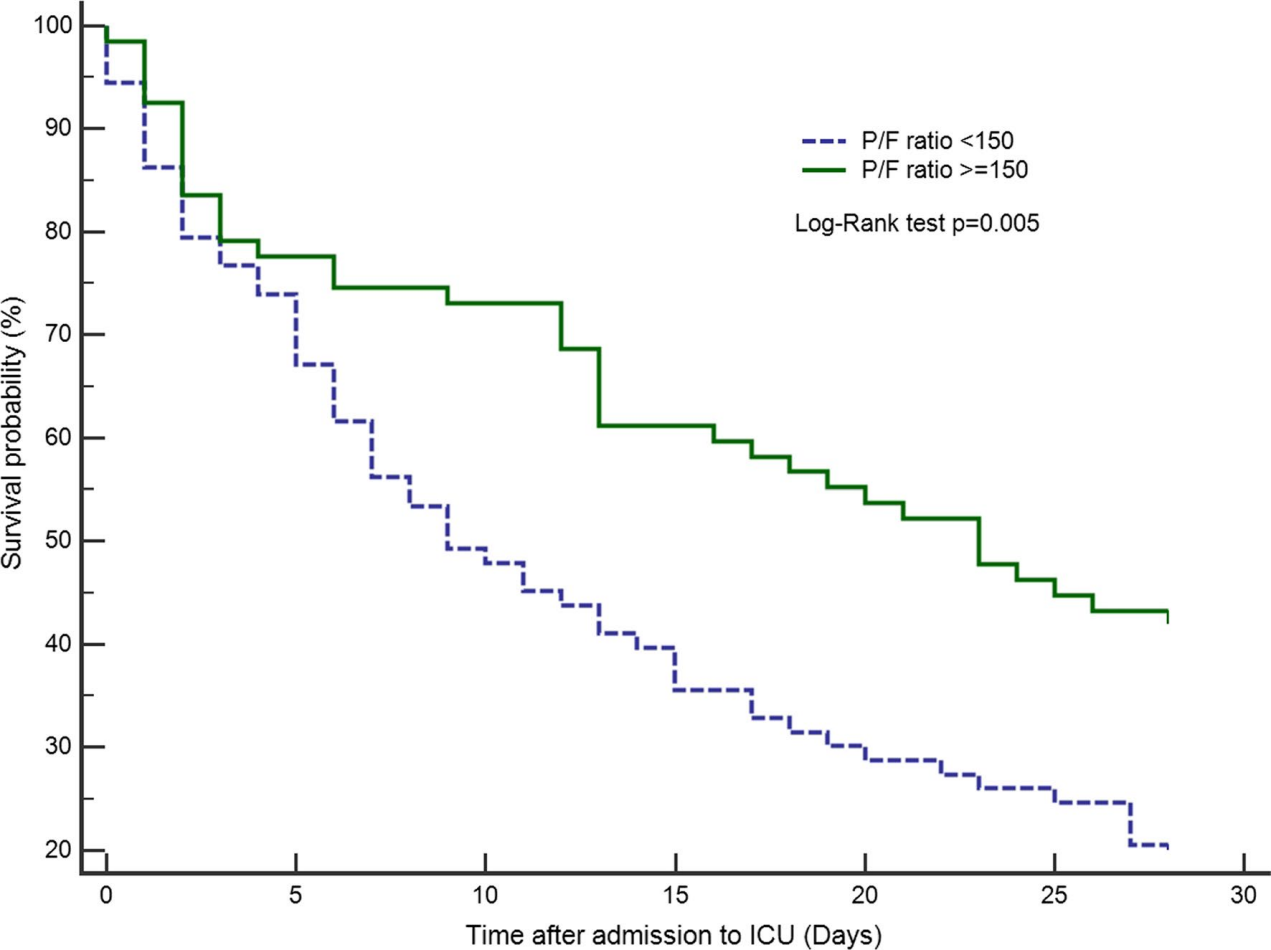
The 28-day mortality according to P/F ratio 150

**Fig. 2:**
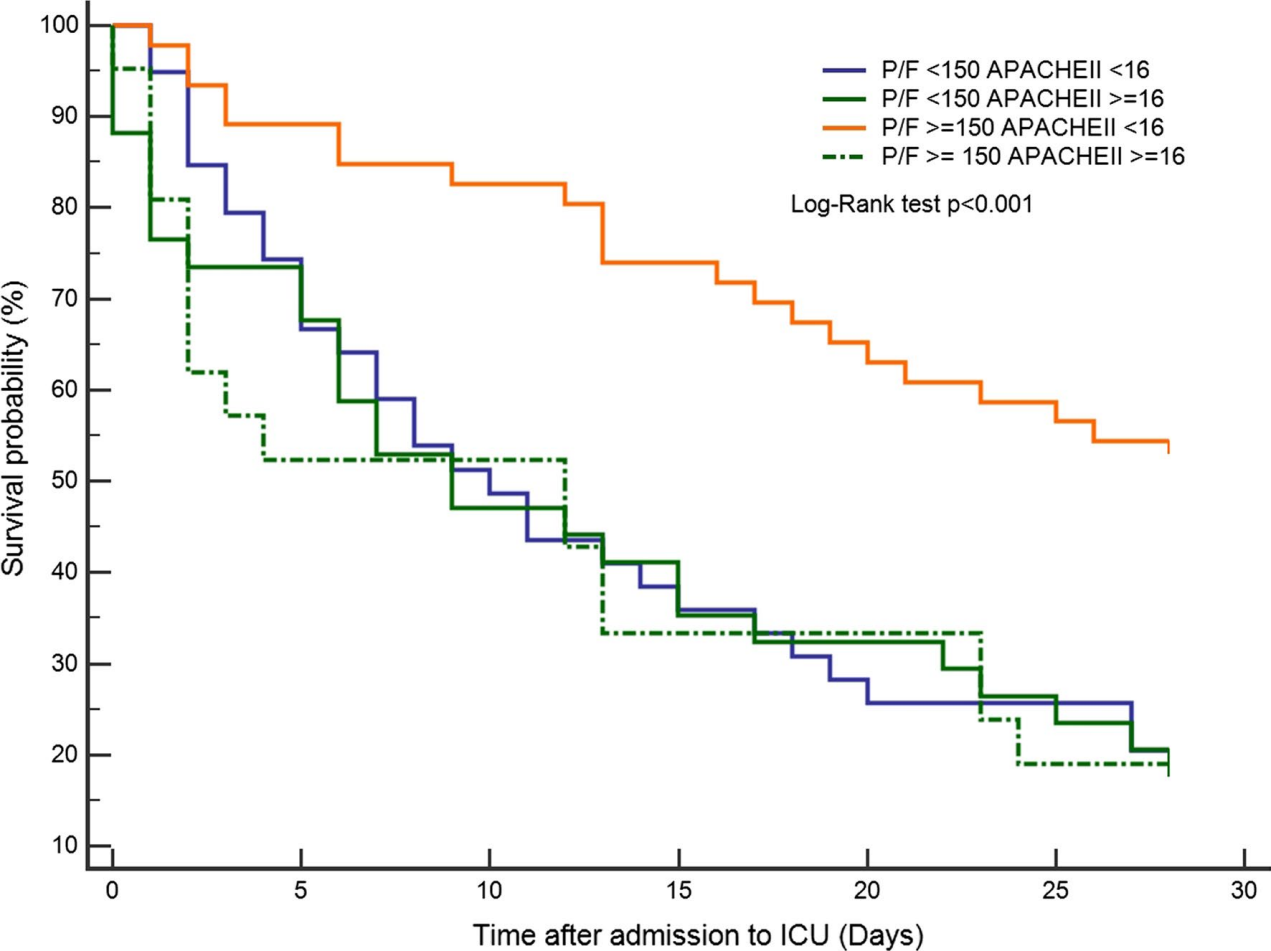
The 28-day mortality according to P/F ratio and APACHE II score

Of the 140 patients, the 28-day and 90-day mortality rates were 68.6% and 88.6%, respectively. Using a P/F ratio ≥ 150 mmHg as the clinical index, Fig. 1 demonstrates that patients with a P/F ratio ≥ 150 had better 28-day survival than those with a P/F ratio < 150 (*p* = 0.005). The difference in survival was significant, especially after the fifth day. Table 2 shows the risk factors associated with 28-day mortality. A P/F ratio ≥ 150 showed a significant lower risk of mortality (OR 0.38; 95% CI 0.17–0.82; *p* = 0.014). Meanwhile, a higher APACHE II score showed a slightly higher association with mortality (OR 1.11; 95% CI 1.03–1.20; *p* = 0.004). Elderly age had little effect on survival, with a marginal hazard ratio (OR 0.96; 95% CI 0.92–0.99; *p* = 0.019). We constructed a receiver operating characteristic (ROC) curve to select the optimal model for predicting risk of mortality. An APACHE II score ≥ 16 was accepted as the cutoff level by a high area under the curve (AUC 0.62) in the ROC curve (Additional file 1: Figure S2A, B, C). By combining the P/F ratio and APACHE II score, those with high P/F ratios and low APACHE II scores during the ICU course had a notable 28-day survival benefit compared to the rest (Fig. 2, *p* < 0.001).

**Table 2:** Univariate and multivariate analysis of 28-day mortality (n = 140)

	Univariate analysis			Multivariate analysis		
	OR	95% CI	*p* value	OR	95% CI	*p* value
Age	0.51	0.24–1.07	0.076	0.96	0.92–0.99	0.019
Smoking history	0.99	0.48–2.02	0.967			
ECOG ≥ 2	0.54	0.26–1.13	0.102			
P/F ≥ 150	0.34	0.16–0.71	0.004	0.38	0.17–0.82	0.014
APACHEII	1.09	1.02–1.16	0.012	1.11	1.03–1.20	0.004
Treatment lines	1.15	0.95–1.40	0.146			
Driver mutation	0.86	0.42–1.76	0.674			
Recent chemotherapy	2.05	0.85–4.93	0.111

### 90-day mortality and predictive factors

**Fig. 3:**
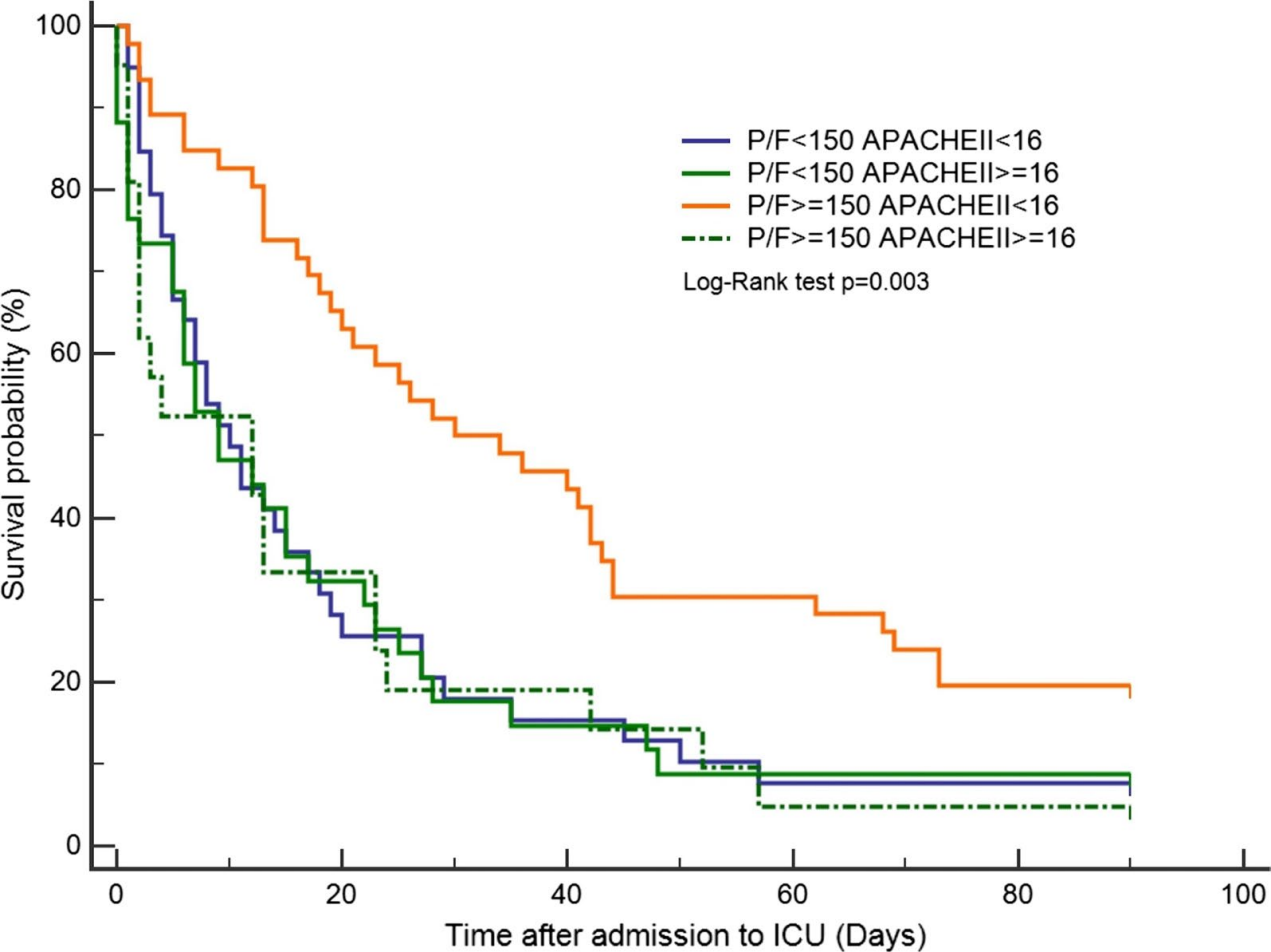
The 90-day mortality according to P/F ratio and APACHE II score

Focusing on the prediction of 90-day mortality, we combined a P/F ratio ≥ 150 and APACHE II score ≥ 16 to demonstrate the survival curve. Those with high P/F ratio ≥ 150 and APACHE II score < 16 during ICU admission still had a 90-day survival benefit (Fig. 3, *p* = 0.003). A comparison of baseline characteristics between 90-day survivors and non-survivors showed no differences in age, sex, smoking history, and performance status. Over two-thirds of 90-day-survivors had driver mutations, and it was significantly different from non-survivors (survivors: 11 (68.8%) vs. non-survivors: 49 (39.5%); *p* = 0.033). Critical status, including APACHE II score, P/F ratio, and blood gas data, showed insignificant differences between 90-day survivors and non-survivors. There was also no specific finding in their recent anticancer treatment and the reasons for ICU admission. Since driver mutation was the only different factor between survivors and non-survivors, we conducted survival analysis by using P/F ratio ≥ 150 and APACHE II score < 16 as the model. The prediction model was significant in those with driver mutations, but not in those without (with driver mutation, *p* = 0.061; without driver mutations, *p* = 0.021).

## Discussion

Cancer patients requiring intensive care support are an important issue in clinical practice [3, 21]. Current guidelines suggest transfer to the ICU for patients with malignancies. Full-code ICU management or time-limited ICU trials should be offered and flexibly adjusted [8, 22]. However, predefined decisions, such as the DNI, are widely accepted in this cohort [19]. Regardless of intubation and mechanical ventilation (MV), these patients may still benefit from NIV [23]. Few studies have focused on the outcome and prognosis of cancer patients with DNI who require intensive care. Whether cancer patients with DNI can benefit from ICU support, and the patient selection criteria, remains debatable [21, 24]. The heterogeneity of cancer patients also brings uncertainty [24]. Patients with lung cancer often have the poorest prognosis in this cohort [2]. Focusing on lung cancer with critical illness, we retrospectively evaluated patients with lung cancer who had DNI orders and discussed the prognostic factors of critical care. We found that a P/F ratio ≥ 150 and an APACHE II score < 16 resulted in the lowest 28-day and 90-day mortality in predefined DNI lung cancer patients. This result offers an objective model for the decision-making of patients with lung cancer and DNI.

The introduction of NIV has had a great impact on critical care [1, 25]. In the LUNG SAFE study, NIV was associated with a higher ICU mortality rate in acute respiratory distress syndrome (ARDS) patients with P/F ratios < 150 mmHg [26]. Evidence also showed that patients with hematologic malignancy and severe respiratory failure were more vulnerable to only NIV support [27–30]. Lin et al. had used initial APACHE III score, FiO2 and number of organ failure as the predictive model for weaning of mechanical ventilation in lung cancer patients [31]. Besides, the 28-day mortality in advanced lung cancer patients who initiate mechanical ventilation at emergent department was poor [32]. However, data focusing on lung cancer patients receiving NIV in the ICU are limited. Our team has pointed out that patients with newly diagnosed or progressive lung cancer have poor outcomes after NIV application [20]. Kim et al. reviewed a cohort of advanced-stage lung cancer patients in critical care and found that a P/F ratio lower than 150 mmHg was independently associated with mortality [14]. In this study, we extended our target group to lung cancer patients with DNIs. Using a P/F ratio of 150 mmHg as the cutoff value, we found that an initial P/F ratio lower than 150 mmHg was predictive of 28-day mortality. However, this clinical feature was not evident after adjusting for the APACHE II score. Patients with a high P/F ratio ≥ 150 mmHg but an APACHE II score ≥ 16 still had no survival benefit. The above findings suggest that a more comprehensive selection criterion for critical care should be applied to lung cancer patients with DNIs.

The P/F ratio and APACHE-II score model predicted not only 28-day mortality, but 90-day mortality as well in patients with lung cancer with DNI. The prognosis showed a significant difference immediately after ICU admission. Other possible factors, such as sex, BMI, and neutropenia, did not differ between survivors and non-survivors. Cancer-related status, such as treatment lines and recent chemotherapy, had no impact on both 28-day and 90-day mortality. Toffart et al. demonstrated that in patients with non-resectable lung cancer requiring intensive care, 3-month survival was associated with the initial performance status and logistic organ dysfunction score [33]. Our findings confirmed that cancer status might also have a limited role in patients with DNI. However, the presence of driver mutations may influence the 90-day survival rate of patients. Our P/F ratio and APACHE II score could only be applied to predict 90-day survival in patients with driver mutations. We assumed that this was due to the feasibility of targeted therapy in a critical care scenario and in patients with marginal status. This is compatible with the finding that patients with lung cancer harboring oncogenic mutations may have different clinical pathways [34]. Several studies have confirmed that tyrosine kinase inhibitors used for patients with mutant lung cancer in critical care units may improve survival [34, 35]. Most of these studies focused on patients with mechanical ventilators [36]. Our study further pointed out that the presence of driver mutations had a possible impact on critical lung cancer patients with DNI. The heterogeneity of patients represents the unmet need to establish a proper classification of these critically ill lung cancer patients.

Our study has several strengths. First, this is the first study to focus on the treatment outcomes of advanced NSCLC patients with DNI in critical settings. As lung cancer accounts for most cancer cases in the ICU and more patients have made predefined do-not-intubate wills before ICU admission, our data were meaningful for clinical decision-making. Second, this was a cohort study conducted in a well-experienced medical center, and general critical care practice may be quite similar. Previous nationwide studies have offered a broad view of this special population, but these studies lacked detailed clinical data. We offered a “closer look” and found that APACHE II and the P/F ratio could be prognostic markers. The results were simple and compatible with the clinical experience. However, our study had some limitations. First, this was a retrospective study with its inherent limitations. We carefully checked the medical records to reduce the rate of missing data. Second, it was a single-center study and lacked of external validation. Besides, the study focused on NSCLC patients with predefined DNI orders. Whether these findings can be applied to other types of lung cancer patients require further investigation. Third, a small proportion of patients did not use NIV. Nevertheless, the distribution was similar between the two subgroups. Fourth, APACHE II scores were generally lower in our cohort. Most of our patients had respiratory failure, but the demands for oxygen or NIV support were not represented in the APACHE II score. Finally, we did not enroll patients who had received immunotherapy before ICU admission. This novel treatment has recently changed the outcome of patients with NSCLC. Whether recent immunotherapy affects the survival of lung cancer patients with DNI requires further study.

## Conclusions

A P/F ratio above 150 and an APACHE II score lower than 16 were prognostic factors for critically ill lung cancer patients with DNI. This prediction could be applied to 90-day survival in patients with driver mutations. This finding was informative during shared decision-making. However, further validation and prospective trials are required.

## Supplementary Information

**Additional file 1: Figure S2A.** ROC curve of APACHE II score. **Figure S2B.** ROC curve of P/F ratio. **Figure S2C.** ROC curve of P/F ratio + APACHEII score. **Table S1.** ICU basic characteristics by 90-day mortality (n = 140). **Figure S3.** 90-day mortality according to PF ratio and APACHEII score by driver mutations. **Table S2.** Multivariate analysis of 90-day mortality. **Figure S4.** Subgroup analysis of P/F ratio >  = 150 and APACHE II < 16 in predicting 28-day mortality.

## Data Availability

The datasets generated and analysed during the current study are not publicly available due to patients’ privacy but are available from the corresponding author on reasonable request.

## Abbreviations

DNI: Do-not-intubate
NIV: Non-invasive ventilation
ICU: Intensive care unit
NSCLC: Non-small cell lung cancer
GCS: Glasgow coma scale
ROC: Receiver operating characteristic
AUC: Area under curve
ARDS: Acute respiratory distress syndrome
