# Supplementary material for: Prognostic factors for advanced lung cancer patients with do-not-intubate order in intensive care unit: a retrospective study
Source: BMC Pulm Med. 2022 Jun 24;22:245. doi: 10.1186/s12890-022-02042-7 (PMC9229461; doi:10.1186/s12890-022-02042-7)
Supplement: Supplementary file 1 — Additional file 1: Figure S2A. ROC curve of APACHE II score. Figure S2B. ROC curve of P/F ratio. Figure S2C. ROC curve of P/F ratio + APACHEII score. Table S1. ICU basic characteristics by 90-day mortality (n = 140). Figure S3. 90-day mortality according to PF ratio and APACHEII score by driver mutations. Table S2. Multivariate analysis of 90-day mortality. Figure S4. Subgroup analysis of P/F ratio > = 150 and APACHE II < 16 in predicting 28-day mortality. [file 12890_2022_2042_MOESM1_ESM.docx]

**Supplementary Figure 1**

**
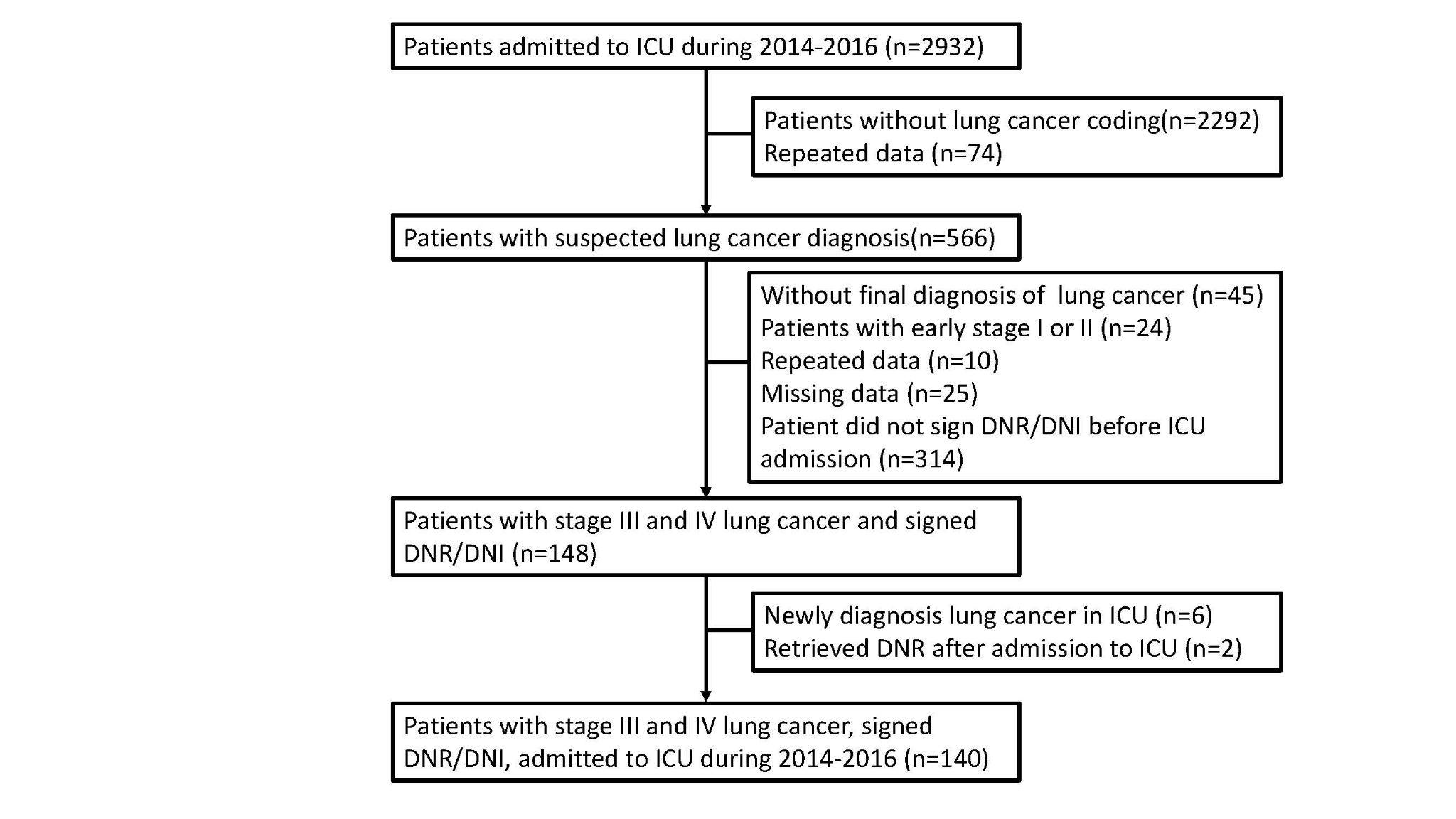
**

**Supplementary Figure 2A ROC curve of APACHE II score**

**
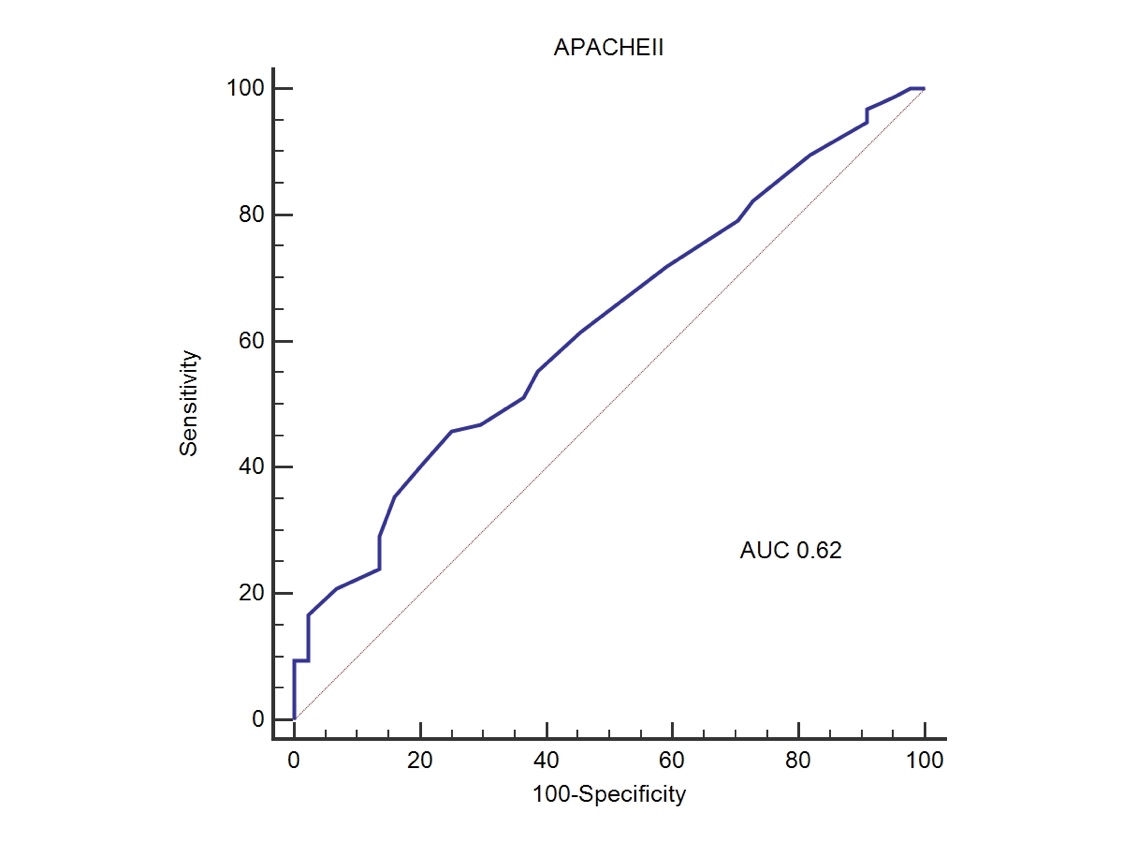
**

**Supplement Figure 2B ROC curve of P/F ratio**


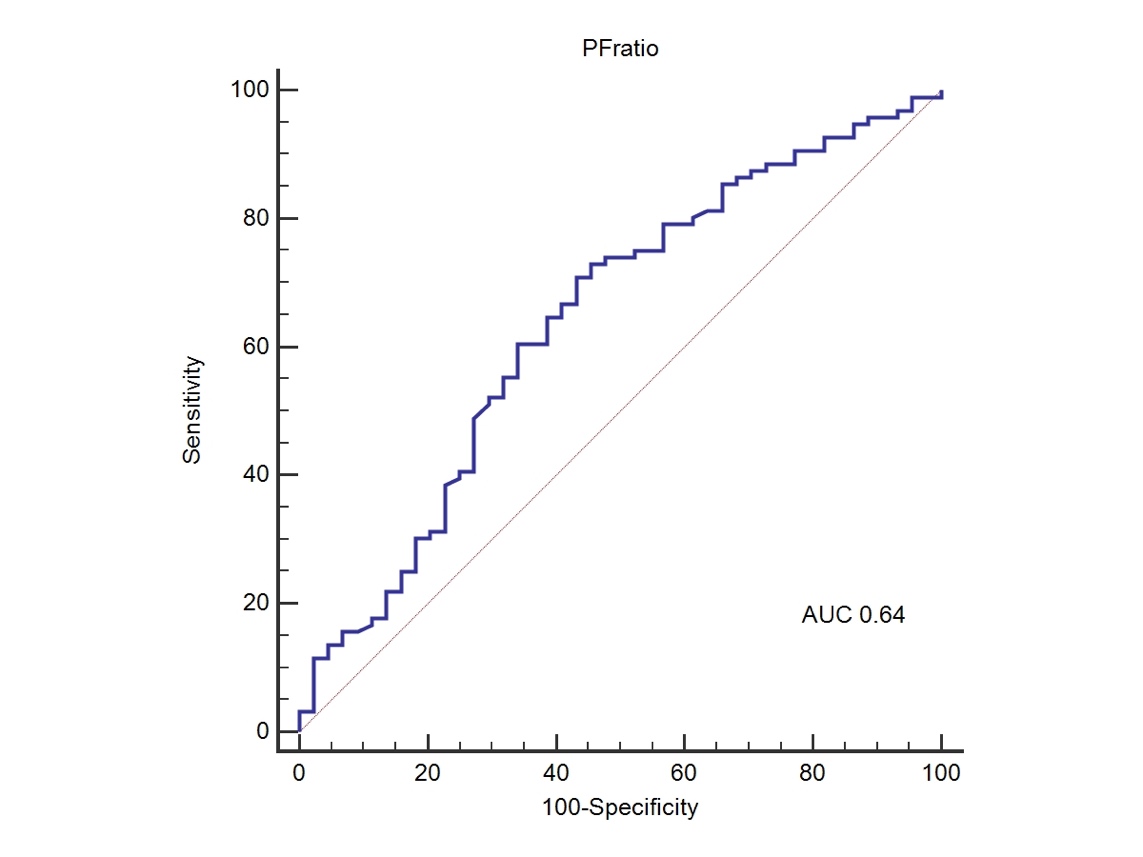


**Supplement Figure 2C ROC curve of P/F ratio + APACHEII score**

**
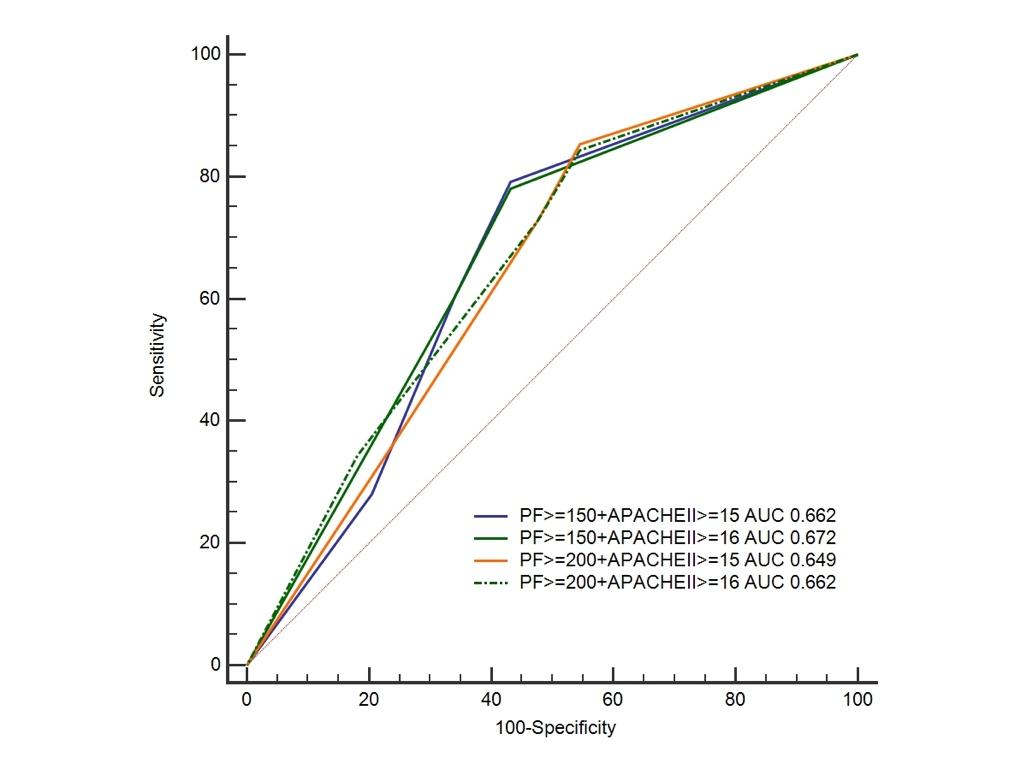
**

**Supplementary Table 1 ICU basic characteristics by 90-day mortality (n=140)**

|  | **All patients (n=140)** | **90 days Survivors (n=16)** | **90 days Non-survivors (n=124)** | **p Value** |
| --- | --- | --- | --- | --- |
| Age (Median, range) | 73 (44-99) | 74 (47-91) | 72 (44-99) | 0.425 |
| BMI (Median, range) | 21 (13-33) | 22 (14-32) | 21 (13-33) | 0.791 |
| Sex |  |  |  |  |
| Male | 87 (62.1) | 9 (56.3) | 78 (62.9) | 0.785 |
| Female | 53 (37.9) | 7 (43.8) | 46 (37.1) |  |
| Smoking history |  |  |  |  |
| Ever smoker | 76 (54.3) | 5 (31.3) | 71 (57.3) | 0.063 |
| Never smoker | 64 (45.7) | 11 (68.8) | 53 (42.7) |  |
| ECOG |  |  |  |  |
| 0-1 | 62 (44.3) | 7 (43.8) | 55 (44.4) | 1.000 |
| >=2 | 78 (55.7) | 9 (56.3) | 69 (55.6) |  |
| Driver mutation | 60 (42.9) | 11 (68.8) | 49 (39.5) | 0.033 |
| Treatment lines | 2 (0-10) | 1 (0-8) | 2 (0-10) | 0.425 |
| APACHE II  (Median, SE) | 14 ($\pm0.66$) | 12 ($\pm1.72$) | 14 ($\pm$0.70) | 0.425 |
| Recent anticancer treatment |  |  |  |  |
| Nil | 52 (37.1) | 6 (37.5) | 46 (37.1) | 0.289 |
| Chemotherapy | 38 (27.1) | 3 (18.8) | 35 (28.2) |  |
| Target therapy | 37 (26.4) | 7 (43.8) | 30 (24.2) |  |
| Radiotherapy | 13 (9.3) | 0 (0) | 13 (10.5) |  |
| GCS | 14 (3-15) | 15 (8-15) | 14 (3-15) | 0.873 |
| PaO_2_/FiO_2_ | 174.2 $\pm$ 104 | 187.8$\pm$ 114.1 | 172.4$\pm$103 | 0.580 |
| PaCO_2_ | 42.5$\pm$16.4 | 41.6$\pm$16.4 | 42.6 $\pm$16.4 | 0.812 |
| pH | 7.40 $\pm$0.11 | 7.41$\pm$0.10 | 7.40$\pm$0.11 | 0.766 |
| Reasons of RCU admission |  |  |  |  |
| Cancer related | 40 (28.6) | 5 (31.3) | 35 (28.2) | 0.855 |
| Treatment related | 15 (10.7) | 1 (6.3) | 14 (11.3) |  |
| Sepsis/infection | 73 (52.1) | 8 (50) | 65 (52.4) |  |
| Cancer related | 40 (28.6) | 2 (12.5) | 10 (8.1) |  |

**Supplementary Figure 3 90-day mortality according to PF ratio and APACHEII score by driver mutations**

**(A)**

**
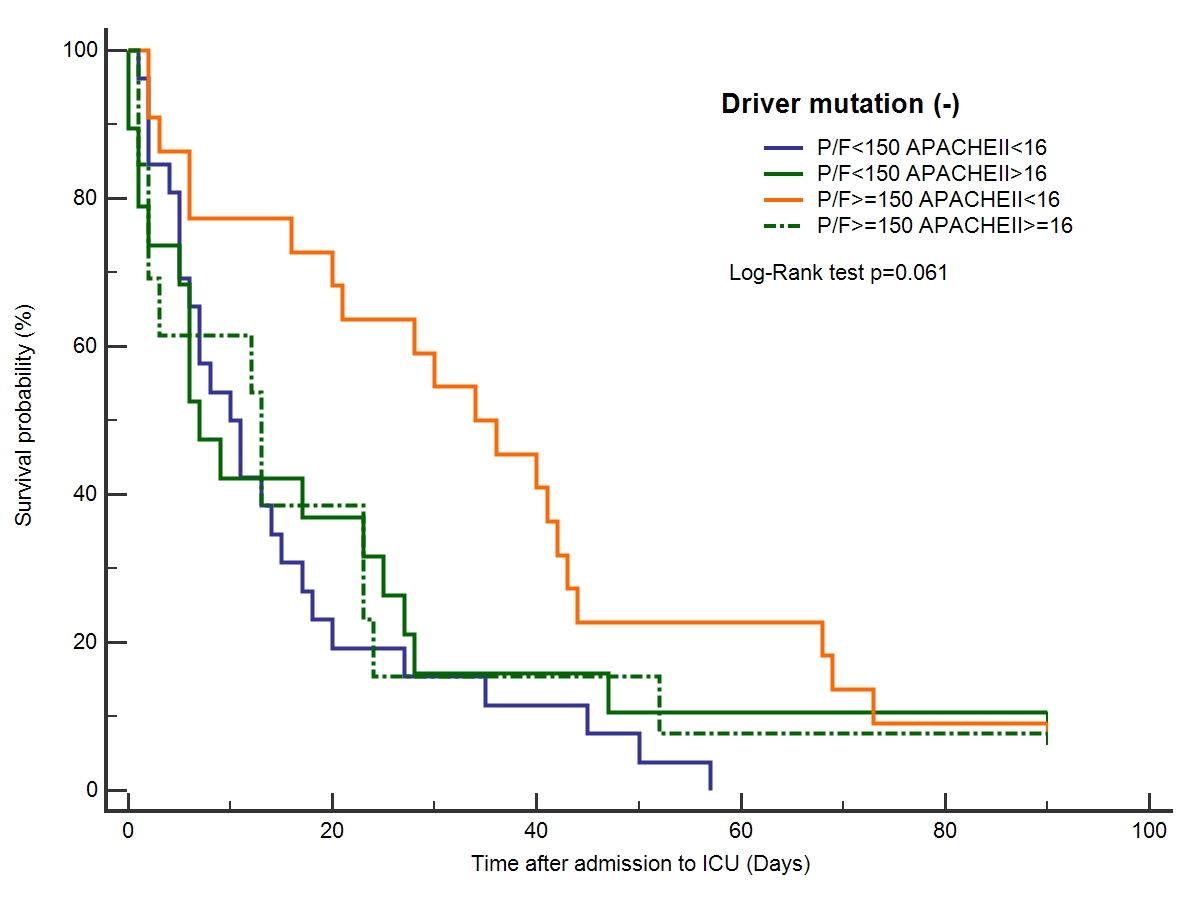
**

**(B)**

**
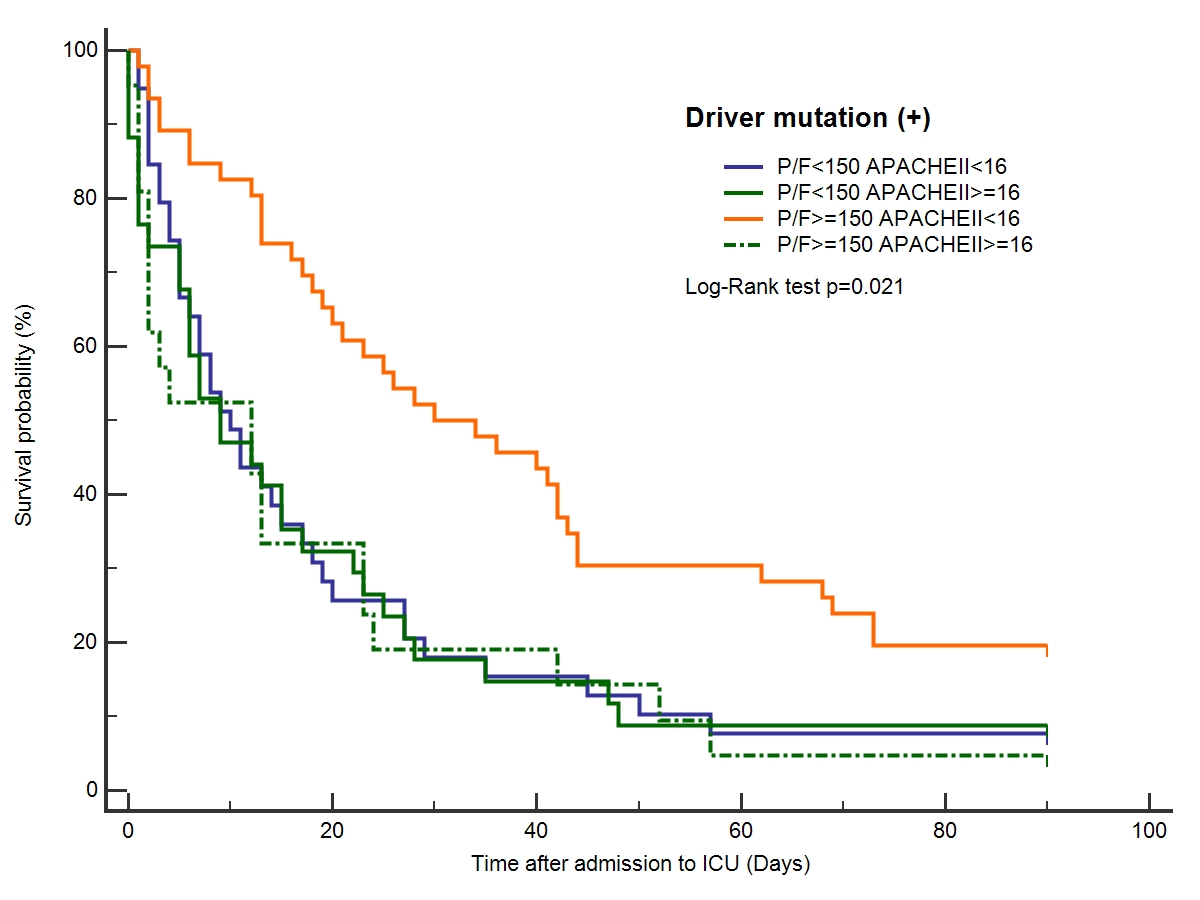
**

**Supplementary Table 2 Multivariate analysis of 90-day mortality**

|  | Univariate analysis | | | Multivariate analysis | | |
| --- | --- | --- | --- | --- | --- | --- |
|  | OR | 95% CI | p value | OR | 95% CI | p value |
| Age | 0.97 | 0.93-1.02 | 0.218 |  |  |  |
| Smoking history | 2.94 | 0.97-8.99 | 0.058 | 2.52 | 0.79-8.09 | 0.119 |
| ECOG ≥ 2 | 0.98 | 0.34-2.79 | 0.963 |  |  |  |
| PF≥150 | 0.51 | 0.18-1.49 | 0.219 |  |  |  |
| APACHEII | 1.09 | 0.99-1.21 | 0.079 | 1.10 | 0.99-1.22 | 0.066 |
| Treatment lines | 1.04 | 0.80-1.35 | 0.771 |  |  |  |
| Driver mutation | 0.30 | 0.10-0.91 | 0.033 | 0.35 | 0.11-1.10 | 0.072 |
| Recent chemotherapy | 1.70 | 0.46-6.35 | 0.427 |  |  |  |

**Supplementary Figure 4 Subgroup analysis of P/F ratio >=150 and APACHE II <16 in predicting 28-day mortality**

**
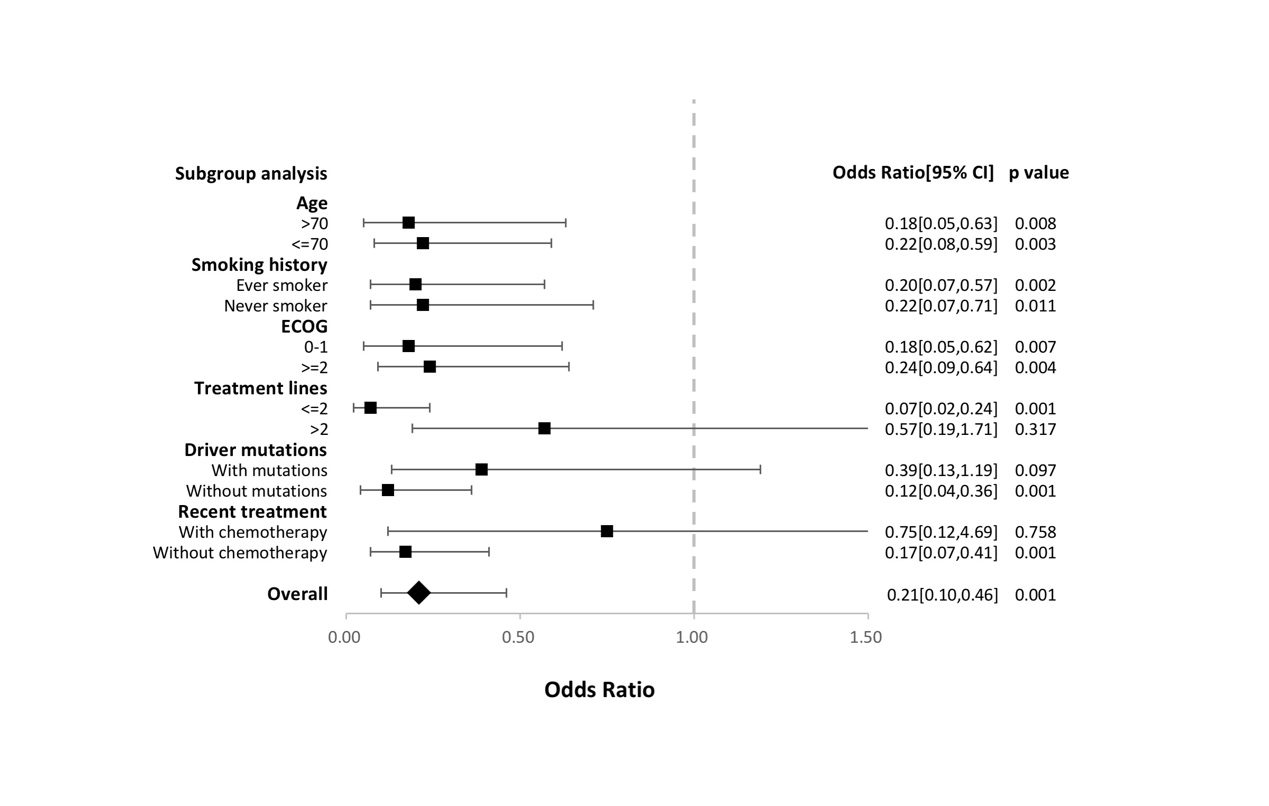
**
